# Variances of Plane Parameters Fitted to Range Data

**DOI:** 10.6028/jres.115.032

**Published:** 2010-12-01

**Authors:** Marek Franaszek

**Affiliations:** National Institute of Standards and Technology,Gaithersburg, MD 20899-0001

**Keywords:** LADAR, nonlinear least squares, variances of fitted parameters

## Abstract

Formulas for variances of plane parameters fitted with Nonlinear Least Squares to point clouds acquired by 3D imaging systems (e.g., LADAR) are derived. Two different error objective functions used in minimization are discussed: the orthogonal and the directional functions. Comparisons of corresponding formulas suggest the two functions can yield different results when applied to the same dataset.

## 1. Introduction

3D imaging systems are line-of-sight instruments that provide range images of objects in a given region of interest, *I*(*ϑ*,*φ*), where *I* denotes the distance from an instrument to a point on a surface of the object and *ϑ* and *φ* are the elevation and the azimuth angles to that point. Usually a point cloud in a Cartesian coordinate system associated with the instrument is derived from the range image. Current 3D imaging systems may collect point clouds containing hundreds of thousands of points within a few seconds [1]. Frequently, these data points are used to model the objects by geometrical primitives that are characterized by attributes such as location, pose, width, height, etc. Numerical values of these attributes may be obtained by fitting a model to the segmented dataset. Here, we discuss the Nonlinear Least Squares (NLS) fitting procedure applied to range data obtained by scanning a plane. Specifically, we address the following problem: how do the uncertainties of range measurements by an instrument propagate to uncertainties of the fitted plane parameters [2]?

Usually, variances of fitted parameters (which are useful for uncertainty analysis) are derived from the Jacobian matrix of a model function used in a given fitting problem. This common approach is based on a linearization of the nonlinear error function near its minimum [3–7]. In this paper we do not follow this path but estimate the variances directly from the sensitivities for which we provide analytical formulas.

## 2. Variances of Fitted Plane Parameters

A plane in a three-dimensional Cartesian coordinate system is defined as a set of points ***P***(*x*, *y*, *z*) satisfying the following equation

(1) P(x,y,z)•w(ϑ,φ)=D

where ***w***(*ϑ*,*φ*) is a unit vector perpendicular to a plane, parameterized by two angles: the elevation *ϑ* (a zero elevation being horizontal) and the azimuth *φ*, and where • stands for the dot product of two vectors. The Cartesian coordinates of ***w***(*ϑ*,*φ*) may be written as

(2) w(ϑ,φ)=[cosϑcosφ,cosϑsinφ,sinϑ].

**Fig. 1:**
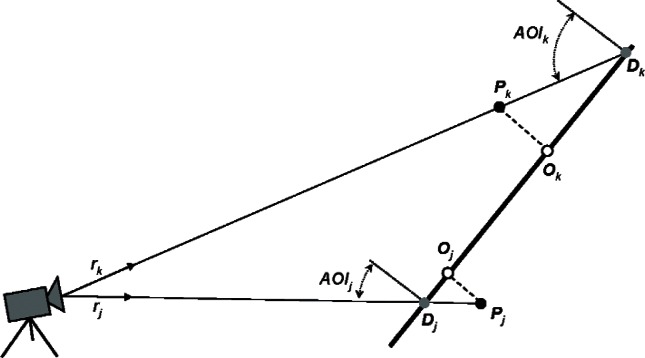
Due to instrument error in measurement of range *r*, experimental points ***P*** do not lie exactly on a plane (thick line). Points ***O*** are perpendicular projections of experimental points on a plane. Points ***D*** are intersections with the plane of rays originating from the instrument and passing through the experimental points. Distances ***PO*** are used in the orthogonal error function *E_O_* while ***PD*** are used in the directional error function *E_D_*. The difference between both functions depends on the Angle of Incidence (AOI).

The absolute value of parameter *D* is the distance from the plane to the origin of the coordinate system, and *D* may be expressed as

(3) $$D=P_{0}(x_{0},y_{0},z_{0})•w\left(\vartheta ,\varphi \right),$$

where ***P***_0_ is any point on a plane. A plane can be fit to the experimental dataset ***P***_{_*_N_*_}_ = {***P****_j_*, *j* = 1,…, *N*}, where *N* denotes the number of points; the goal being to calculate the numerical values of the three parameters defining the plane: *ϑ*, *φ* and *D*. Within the framework of the Least Squares method, the fit parameters are obtained by minimizing the error function

(4) $$E(\vartheta ,\varphi ,D,P_{\left\{N\right\}})=\frac{1}{N}\sum_{j=1}^{N}E_{j}^{2}(\vartheta ,\varphi ,D,P_{j}),$$

where *E_j_* is the distance between the experimental point ***P****_j_* and its corresponding “theoretical point.” Different definitions of the theoretical point yield different error functions. In this paper we study two error functions: the orthogonal error function *E_O_* and the directional error function *E_D_*, as explained in Fig. 1 and in the next two sections.

It is not surprising that due to nonlinear dependence of the normal vector ***w*** on both angles *ϑ* and *φ*, plane fitting requires nonlinear minimization. However, as is shown in the next two sections, for both error functions *E_O_* and *E_D_* the distance *E_j_* depends linearly on the third parameter *D*. Therefore, *D* may be explicitly expressed as a function of both angles (*ϑ*, *φ*) and ***P***_{_*_N_*_}_ from the condition

(5) $$\frac{\partial E}{\partial D}(\vartheta ,\varphi ,D,P_{\left\{N\right\}})=0.$$

For any error function *E* defined by Eq. (4) with *E_j_* depending linearly on *D*, the linear parameter can be expressed as a function of the remaining non-linear parameters and dataset ***P***_{_*_N_*_}_

(6) $$D=D(\vartheta ,\varphi ,P_{\left\{N\right\}}).$$

When the error function *E* reaches its minimum at [ϑ^*^, φ^*^, D^*^], a gradient of the function has to be zero. This implies that the original 3D search space may be reduced to a 2D space and the error function may be re-written as

(7) $$E(\vartheta ,\varphi ,P_{\left\{N\right\}})=\frac{1}{N}\sum_{j=1}^{N}E_{j}^{2}(\vartheta ,\varphi ,P_{j}),$$

the minimum of *E* being located at

(8) $$\vartheta^{*}=\vartheta^{*}(P_{\left\{N\right\}}),\;\varphi^{*}=\varphi^{*}(P_{\left\{N\right\}}),\;D^{*}=D(\vartheta^{*},\varphi^{*},P_{\left\{N\right\}}).$$

The variances of the fitted plane parameters var(ϑ^*^) and var(φ^*^) may be calculated following the same general approach developed for fitting a sphere to range data [8], [9]. In the current study, the same assumption is made as in the previous studies: for the 3D imaging systems relevant to this study, the uncertainty in the range measurement is typically much larger than the uncertainty in the angular measurements. Thus, an acquired point ***P****_j_* can be expressed as

(9a) $$P_{j}=r_{j}p_{j}(\vartheta_{j},\varphi_{j}),$$

where *r_j_* is a range measured at bearings (*ϑ_j_*, *φ_j_*) and ||***P****_j_*|| = *r_j_*. In this approximation the bearings are treated as noise-free control variables and a unit vector ***p****_j_* is defined as

(9b) $$p_{j}(\vartheta_{j},\varphi_{j})=[\cos\vartheta_{j}\cos\varphi_{j},\cos\vartheta_{j}\sin\varphi_{j},\sin\vartheta_{j}].$$

Note that for other types of instruments, for example Coordinate Measuring Machines (CMM), the above assumption may not be valid and the formulas for variances of fitted plane parameters developed in this paper may not be applicable. In addition, it is assumed that the correlation in the measured ranges *r_j_* and *r_k_* is negligible for any *j* ≠ *k*. When both assumptions are valid, the variances of the fitted plane parameters may be estimated by applying to Eq. (8) the uncertainty propagation formula [2]

(10a) $$\mathrm{var}(\vartheta^{*})\approx {\sum_{j=1}^{N}\left[\frac{\partial \vartheta^{*}(P_{\left\{N\right\}})}{\partial r_{j}}\right]}^{2}\mathrm{var}(r_{j}),$$

(10b) $$\mathrm{var}(\varphi^{*})\approx {\sum_{j=1}^{N}\left[\frac{\partial \varphi^{*}(P_{\left\{N\right\}})}{\partial r_{j}}\right]}^{2}\mathrm{var}(r_{j}),$$

and the covariance may be estimated as

(10c) $$\mathrm{cov}(\vartheta^{*},\varphi^{*})\approx \sum_{j=1}^{N}\left[\frac{\partial \vartheta^{*}(P_{\left\{N\right\}})}{\partial r_{j}}\right]\left[\frac{\partial \varphi^{*}(P_{\left\{N\right\}})}{\partial r_{j}}\right]\mathrm{var}(r_{j}).$$

The variance of the third parameter D^*^ may be calculated from the uncertainty propagation formula [2] applied to a general function *D*(*ϑ*,*φ*,***P***_{_*_N_*_}_) defined in Eq. (6),

(11) $$\mathrm{var}(D^{*})=\sum_{j=1}^{N}{\left[\frac{\partial D}{\partial \vartheta }\frac{\partial \vartheta^{*}}{\partial r_{j}}+\frac{\partial D}{\partial \varphi }\frac{\partial \varphi^{*}}{\partial r_{j}}+\frac{\partial D}{\partial r_{j}}\right]}^{2}\mathrm{var}(r_{j})$$

where the derivatives of *D* are calculated at (ϑ^*^, φ^*^, ***P***_{_*_N_*_}_).

The individual sensitivities $\frac{\partial \vartheta^{*}}{\partial r_{j}},\frac{\partial \varphi^{*}}{\partial r_{j}}$ used in Eqs. (10) and (11) may be calculated as in [8] by solving for each *j* the following 2 × 2 system of linear equations

(12) $$H(\vartheta^{*},\varphi^{*},P_{\left\{N\right\}})S_{j}(P_{\left\{N\right\}})=-V_{j}(\vartheta^{*},\varphi^{*},P_{\left\{N\right\}}),$$

where the vectors ***S****_j_* and ***V****_j_* are defined as

(13a) $$S_{j}(P_{\left\{N\right\}})=\left[\begin{array}{c} \frac{\partial \vartheta^{*}(P_{\left\{N\right\}})}{\partial r_{j}}\\ \frac{\partial \varphi^{*}(P_{\left\{N\right\}})}{\partial r_{j}} \end{array}\right],$$

(13b) $$V_{j}(\vartheta ,\varphi ,P_{\left\{N\right\}})=\left[\begin{array}{c} \frac{\partial^{2}E(\vartheta ,\varphi ,P_{\left\{N\right\}})}{\partial r_{j}\partial \vartheta }\\ \frac{\partial^{2}E(\vartheta ,\varphi ,P_{\left\{N\right\}})}{\partial r_{j}\partial \varphi } \end{array}\right].$$

The matrix ***H*** is the Hessian of the error function *E*(*ϑ*, *φ*, ***P***_{_*_N_*_}_)

(14) $$H(\vartheta ,\varphi ,P_{\left\{N\right\}})=\left[\begin{array}{cc} \frac{\partial^{2}E}{\partial \vartheta^{2}} & \frac{\partial^{2}E}{\partial \vartheta \partial \varphi }\\ \frac{\partial^{2}E}{\partial \varphi \partial \vartheta } & \frac{\partial^{2}E}{\partial \varphi^{2}} \end{array}\right].$$

These general formulas are now applied to two specific error functions: the orthogonal error function *E_O_* and the directional error function *E_D_*.

## 3. Orthogonal Fitting

For the orthogonal plane fitting (see Fig. 1) the theoretical point ***O****_j_* corresponding to the experimental point ***P****_j_* is defined as the orthogonal projection of ***P****_j_* on a plane. Thus, Eq. (4) takes the form

(15) $$E_{o}(\vartheta ,\varphi ,D,P_{\left\{N\right\}})=\frac{1}{N}\sum_{j=1}^{N}{\left[w(\vartheta ,\varphi )•P_{j}-D\right]}^{2}.$$

Applying condition (5), Equation (6) can be expressed as

(16) $$D(\vartheta ,\varphi ,P_{\left\{N\right\}})=w(\vartheta ,\varphi )•\left(\frac{1}{N}\sum_{j=1}^{N}P_{j}\right)=w•P_{0},$$

where ***P***_0_ here is the centroid of all experimental points ***P***_{_*_N_*_}_. This condition states that the plane fitted with the orthogonal error function has to contain the centroid, ***P***_0_. Defining a scalar product *d_j_* of two vectors ***w*** and ***p****_j_* given by Eqs. (2) and (9b)

(17) $$d_{j}(\vartheta ,\varphi )=w(\vartheta ,\varphi )•p_{j}(\vartheta_{j},\varphi_{j}),$$

then Eq. (16) for the parameter *D* when using Eqs. (17) and (9a) can be rewritten as

(18) $$D(\vartheta ,\varphi ,P_{\left\{N\right\}})=\frac{1}{N}\sum_{j=1}^{N}d_{j}(\vartheta ,\varphi )r_{j},$$

where *r_j_* is a measured range. In this notation, Eq. (7) describing the error function in the reduced 2D search space of angles (*ϑ*, *φ*) can be written as

(19a) $$E_{o}(\vartheta ,\varphi ,P_{\left\{N\right\}})=\frac{1}{N}\sum_{j=1}^{N}{\left(w•U_{j}\right)}^{2},$$

where

(19b) $$U_{j}=P_{j}-P_{0}.$$

Then, the elements of the gradient of the error function ∇*E_O_* defined in Eq. (19a) can be calculated as

(20a) $$\frac{\partial E_{o}}{\partial \vartheta }(\vartheta ,\varphi ,P_{\left\{N\right\}})=\frac{2}{N}\sum_{j=1}^{N}(w•U_{j})\left(\frac{\partial w}{\partial \vartheta }•U_{j}\right),$$

(20b) $$\frac{\partial E_{o}}{\partial \varphi }(\vartheta ,\varphi ,P_{\left\{N\right\}})=\frac{2}{N}\sum_{j=1}^{N}(w•U_{j})\left(\frac{\partial w}{\partial \varphi }•U_{j}\right),$$

while the elements of the Hessian matrix ***H*** defined in Eq. (14) are

(21a) $$\begin{array}{l} \frac{\partial^{2}E_{o}}{\partial \vartheta^{2}}(\vartheta ,\varphi ,P_{\left\{N\right\}})=\\ \;\frac{2}{N}\sum_{j=1}^{N}\left[{\left(\frac{\partial w}{\partial \vartheta }•U_{j}\right)}^{2}+(w•U_{j})\left(\frac{\partial^{2}w}{\partial \vartheta^{2}}•U_{j}\right)\right], \end{array}$$

(21b) $$\begin{array}{l} \frac{\partial^{2}E_{o}}{\partial \varphi^{2}}(\vartheta ,\varphi ,P_{\left\{N\right\}})=\\ \;\frac{2}{N}\sum_{j=1}^{N}\left[{\left(\frac{\partial w}{\partial \varphi }•U_{j}\right)}^{2}+(w•U_{j})\left(\frac{\partial^{2}w}{\partial \varphi^{2}}•U_{j}\right)\right], \end{array}$$

(21c) $$\begin{array}{l} \frac{\partial^{2}E_{o}}{\partial \vartheta \partial \varphi }(\vartheta ,\varphi ,P_{\left\{N\right\}})=\\ \;\frac{2}{N}\sum_{j=1}^{N}\left[\left(\frac{\partial w}{\partial \vartheta }•U_{j}\right)\left(\frac{\partial w}{\partial \varphi }•U_{j}\right)+(w•U_{j})\left(\frac{\partial^{2}w}{\partial \vartheta \partial \varphi }•U_{j}\right)\right]. \end{array}$$

Finally, the elements of vector ***V****_j_* defined in Eq. (13b) can be obtained by differentiating with respect to *r_j_* the elements of the gradient ∇*E_O_* given by Eqs. (20a) and (20b). Taking into account the definition of vector ***U****_j_* given by Eq. (19b) and the definition of centroid ***P***_0_ of all points ***P****_j_* as well the dependence of ***P****_j_* on *r_j_* given by Eq. (9a), the elements of vector ***V****_j_* can be evaluated as

(22a) $$\begin{array}{l} \frac{\partial^{2}E_{o}}{\partial r_{j}\partial \vartheta }(\vartheta ,\varphi ,P_{\left\{N\right\}})=\\ \frac{2}{N}\sum_{k=1}^{N}\frac{\partial U_{k}}{\partial r_{j}}•\left[\left(\frac{\partial w}{\partial \vartheta }•U_{k}\right)w+(w•U_{k})\frac{\partial w}{\partial \vartheta }\right], \end{array}$$

(22b) $$\begin{array}{l} \frac{\partial^{2}E_{o}}{\partial r_{j}\partial \varphi }(\vartheta ,\varphi ,P_{\left\{N\right\}})=\\ \frac{2}{N}\sum_{k=1}^{N}\frac{\partial U_{k}}{\partial r_{j}}•\left[\left(\frac{\partial w}{\partial \varphi }•U_{k}\right)w+(w•U_{k})\frac{\partial w}{\partial \varphi }\right], \end{array}$$

where

(22c) $$\frac{\partial U_{k}}{\partial r_{j}}=\left(\delta_{j,k}-\frac{1}{N}\right)p_{k},$$

*δ_j,k_* is the Kronecker delta, and ***p****_k_* is the unit vector defined in Eq. (9b). First and second order derivates of the vector ***w***(*ϑ*,*φ*), defined in Eq. (2), are provided in Appendix A, Eqs. (A1–A5). Once the matrix ***H*** and vectors ***V****_j_* are known, the sensitivity vectors ***S****_j_* can be calculated for every *j* by solving the 2 × 2 system of linear Eq. (12). The variances of the fitted angles var(ϑ^*^) and var(φ^*^) and the covariance cov(ϑ^*^,φ^*^) can then be determined from Eqs. (10a, b, c). The variance of the third parameter D^*^, defined in Eqs. (16–18), can be now evaluated from Eq. (11) using the following equations:

(23a) $$\frac{\partial D}{\partial \vartheta }=\frac{\partial w}{\partial \vartheta }•P_{0},$$

(23b) $$\frac{\partial D}{\partial \varphi }=\frac{\partial w}{\partial \varphi }•P_{0},$$

(23c) $$\frac{\partial D}{\partial r_{j}}=\frac{1}{N}d_{j},$$

where the derivatives of *D* are calculated at [ϑ^*^,φ^*^, ***P***_{_*_N_*_}_].

## 4. Directional Fitting

For the directional plane fitting (see Fig. 1) the theoretical point ***D****_j_* corresponding to the experimental point ***P****_j_* is defined as an intersection of a ray originating from the instrument through ***P****_j_* with the plane

(24a) $$D_{j}=t_{j}P_{j}$$

where a parameter *t_j_* has its value close to 1, and the theoretical points satisfy Eq. (1) of the plane

(24b) $$D_{j}(x_{j},y_{j},z_{j})•w(\vartheta ,\varphi )=D.$$

The distance *E_j_* in Eq. (4) is the Euclidian norm and the directional error function *E_D_* can thus be written as

(25) $$E_{D}(\vartheta ,\varphi ,D,P_{\left\{N\right\}})=\frac{1}{N}\sum_{j=1}^{N}{\left\|D_{j}-P_{j}\right\|}^{2}$$

where the parameter *t_j_* can be calculated from Eq. (24) using the *d_j_* defined in Eq. (17)

(26) $$t_{j}=\frac{D}{r_{j}d_{j}}$$

if *d_j_* is different from zero, i.e., if the vector ***p****_j_* is not orthogonal to ***w***. Two vectors ***p****_j_* and ***w*** are orthogonal only if the corresponding AOI = ± 90°, which causes the theoretical point ***D****_j_* to be undefined. For all other AOIs, *t_j_* can be calculated and substituted into Eq. (24a). Then, using Eq. (9a) and the fact that *r_j_* = ||***P****_j_*||, Eq. (25) yields the following expression for the directional error function

(27) $$E_{D}(\vartheta ,\varphi ,D,P_{\left\{N\right\}})=\frac{1}{N}\sum_{j=1}^{N}{\left(\frac{D}{d_{j}}-r_{j}\right)}^{2}.$$

Applying condition (5) to Eq. (27), Eq. (6) can be expressed as

(28) $$D(\vartheta ,\varphi ,P_{\left\{N\right\}})=\frac{\sum_{j=1}^{N}r_{j}d_{j}^{-1}}{\sum_{j=1}^{N}d_{j}^{-2}}.$$

In this notation, Eq. (7) describing the error function in the reduced 2D search space of angles (*ϑ*,*φ*) can be written as

(29) $$E_{D}(\vartheta ,\varphi ,P_{\left\{N\right\}})=\frac{1}{N}\sum_{j=1}^{N}{\left(\frac{D(\vartheta ,\varphi ,P_{\left\{N\right\}})}{d_{j}(\vartheta ,\varphi )}-r_{j}\right)}^{2}.$$

Using the following auxiliary functions

(30a) $$\begin{array}{l} A_{\vartheta ,j}(\vartheta ,\varphi ,P_{\left\{N\right\}})=\\ \frac{\partial }{\partial \vartheta }\left(\frac{D}{d_{j}}-r_{j}\right)=\left(d_{j}\frac{\partial D}{\partial \vartheta }-D\frac{\partial d_{j}}{\partial \vartheta }\right)d_{j}^{-2}, \end{array}$$

(30b) $$\begin{array}{l} A_{\varphi ,j}(\vartheta ,\varphi ,P_{\left\{N\right\}})=\\ \frac{\partial }{\partial \varphi }\left(\frac{D}{d_{j}}-r_{j}\right)=\left(d_{j}\frac{\partial D}{\partial \varphi }-D\frac{\partial d_{j}}{\partial \varphi }\right)d_{j}^{-2}, \end{array}$$

it is possible to calculate the elements of the gradient of the error function ∇*E_D_*

(31a) $$\frac{\partial E_{D}}{\partial \vartheta }(\vartheta ,\varphi ,P_{\left\{N\right\}})=\frac{2}{N}\sum_{j=1}^{N}\left(\frac{D}{d_{j}}-r_{j}\right)A_{\vartheta ,j},$$

(31b) $$\frac{\partial E_{D}}{\partial \varphi }(\vartheta ,\varphi ,P_{\left\{N\right\}})=\frac{2}{N}\sum_{j=1}^{N}\left(\frac{D}{d_{j}}-r_{j}\right)A_{\varphi ,j},$$

and the elements of the Hessian matrix ***H*** defined in Eq. (14)

(32a) $$\frac{\partial^{2}E_{D}}{\partial \vartheta^{2}}(\vartheta ,\varphi ,P_{\left\{N\right\}})=\frac{2}{N}\sum_{j=1}^{N}\left[A_{\vartheta ,j}^{2}+\left(\frac{D}{d_{j}}-r_{j}\right)\frac{\partial A_{\vartheta ,j}}{\partial \vartheta }\right],$$

(32b) $$\frac{\partial^{2}E_{D}}{\partial \varphi^{2}}(\vartheta ,\varphi ,P_{\left\{N\right\}})=\frac{2}{N}\sum_{j=1}^{N}\left[A_{\varphi ,j}^{2}+\left(\frac{D}{d_{j}}-r_{j}\right)\frac{\partial A_{\varphi ,j}}{\partial \varphi }\right],$$

(32c) $$\frac{\partial^{2}E_{D}}{\partial \varphi \partial \vartheta }(\vartheta ,\varphi ,P_{\left\{N\right\}})=\frac{2}{N}\sum_{j=1}^{N}\left[A_{\vartheta ,j}A_{\varphi ,j}+\left(\frac{D}{d_{j}}-r_{j}\right)\frac{\partial A_{\vartheta ,j}}{\partial \varphi }\right].$$

The derivatives $\frac{\partial D}{\partial \vartheta }$ and $\frac{\partial D}{\partial \varphi }$ in Eqs. (30a,b) are calculated in the Appendix B, Eqs. (B17, B18), $\frac{\partial d_{j}}{\partial \vartheta }$ and $\frac{\partial d_{j}}{\partial \varphi }$ in (A6, A7), $\frac{\partial A_{\vartheta ,j}}{\partial \vartheta }$, $\frac{\partial A_{\varphi ,j}}{\partial \varphi }$ and $\frac{\partial A_{\vartheta ,j}}{\partial \varphi }$ in (C1–C3). Finally, the elements of vector ***V****_j_* defined in Eq. (13b) can be expressed as

(33a) $$\begin{array}{l} \frac{\partial^{2}E_{D}}{\partial r_{j}\partial \vartheta }(\vartheta ,\varphi ,P_{\left\{N\right\}})=\\ \frac{2}{N}\sum_{k=1}^{N}\left[\left(\frac{\partial D}{\partial r_{j}}d_{k}^{-1}-\delta_{j,k}\right)A_{\vartheta ,k}+\left(\frac{D}{d_{k}}-r_{k}\right)\frac{\partial A_{\vartheta ,k}}{\partial r_{j}}\right], \end{array}$$

(33b) $$\begin{array}{l} \frac{\partial^{2}E_{D}}{\partial r_{j}\partial \varphi }(\vartheta ,\varphi ,P_{\left\{N\right\}})=\\ \frac{2}{N}\sum_{k=1}^{N}\left[\left(\frac{\partial D}{\partial r_{j}}d_{k}^{-1}-\delta_{j,k}\right)A_{\varphi ,k}+\left(\frac{D}{d_{k}}-r_{k}\right)\frac{\partial A_{\varphi ,k}}{\partial r_{j}}\right], \end{array}$$

where the derivative $\frac{\partial D}{\partial r_{j}}$ is calculated in Appendix B, Eq. (B22), $\frac{\partial A_{\vartheta ,k}}{\partial r_{j}}$ and $\frac{\partial A_{\varphi ,k}}{\partial r_{j}}$ in Eqs. (C4, C5), and *δ_j,k_* is the Kronecker delta. Once the matrix ***H*** and vectors ***V****_j_* are known, the sensitivity vectors ***S****_j_* can be calculated for every *j* by solving a 2 × 2 system of linear equations (12). The variances of fitted angles var(ϑ^*^) and var(φ^*^) and the covariance cov(ϑ^*^,φ^*^) can then be determined from Eqs. (10a, b, c). The variance of the third parameter D^*^, defined in Eq. (28), can now be evaluated by substituting in Eq. (11) the Eqs. (B17, B18, B22), for the derivatives $\frac{\partial D}{\partial \vartheta }$, $\frac{\partial D}{\partial \varphi }$ and $\frac{\partial D}{\partial r_{j}}$ calculated at [ϑ*,φ*, ***P***_{_*_N_*_}_].

## 5. Discussion

Figure 1 shows that the orthogonal plane fitting behaves differently from the directional fitting. The difference between orthogonal and directional fitting depends on the Angle of Incidence (AOI) of the laser beam. For AOI approaching 90°, the optimal value of the orthogonal error function *E_O_*(ϑ^*^,φ^*^, ***P***_{_*_N_*_}_) is decreasing, even when the uncertainty of the measured ranges *r_j_* is large. The optimal value of the error function is usually interpreted as a gauge of noise level in experimental data (assuming that a right model is fitted to the data). For 3D imaging systems, due to a divergence of a laser beam, range measurements collected for large AOI have large uncertainty. Thus, the behavior of *E_O_* is in a sharp contrast with the common experimental observation. The directional fitting is free of this flaw and a residual value of the directional error function *E_D_*(ϑ^*^,φ^*^, ***P***_{_*_N_*_}_) correctly estimates a level of noise in the acquired experimental dataset ***P***_{_*_N_*_}_ for any AOI. When *E_O_* is minimized, the sensitivities of the fitted angles $\frac{\partial \vartheta^{*}}{\partial r_{j}}$ and $\frac{\partial \varphi^{*}}{\partial r_{j}}$ may be underestimated for large AOI. The flawed sensitivities entered in the Eqs. (10) and (11) will cause an underestimation of the variances of the plane parameters fitted with the orthogonal function for large AOI. For small AOI, the difference between *E_O_* and *E_D_* is diminishing and both error functions are expected to provide correct estimates for the variances of fitted parameters.

Individual sensitivities ***S****_j_* of fitted angles are calculated from Eq. (12). The vector ***V****_j_* on the right hand side of this equation behaves differently for the orthogonal and the directional error function, see Eqs. (D1, 2) and (D7, 8) in the Appendix D. This may cause a much larger variability of ***S****_j_* calculated for the orthogonal fitting and a poorer estimate of variances of fitted parameters than for the directional fitting.

As was already pointed out, a plane fitted by minimizing the orthogonal error function has to contain the centroid ***P***_0_ of the acquired points ***P***_{_*_N_*_}_, see Eq. (16). Directional fitting does not have this constraint. Thus, a minimization of two error functions discussed in this paper may lead to different results.

## 6. Conclusions

In this paper we derived formulas for the variances (which are useful for uncertainty analysis) of plane parameters fitted to a dataset acquired with 3D imaging systems. Two error functions were investigated: the orthogonal and the directional error function. Comparison of corresponding formulas suggests the two functions may yield different results when applied to the same range data. However, in order to quantify the anticipated difference, laboratory experiments and computer simulations are needed.

## 7. Appendix A

From the definition of ***w*** (*ϑ*,*φ*) in Eq. (2), the following derivates can be calculated

(A1) $$\frac{\partial w}{\partial \vartheta }=[-\sin\vartheta \cos\varphi ,-\sin\vartheta \sin\varphi ,\cos\vartheta ]$$

(A2) $$\frac{\partial w}{\partial \varphi }=[-\cos\vartheta \sin\varphi ,\cos\vartheta \cos\varphi ,0]$$

(A3) $$\frac{\partial^{2}w}{\partial \vartheta^{2}}=-w$$

(A4) $$\frac{\partial^{2}w}{\partial \varphi^{2}}=[-\cos\vartheta \cos\varphi ,-\cos\vartheta \sin\varphi ,0]$$

(A5) $$\frac{\partial^{2}w}{\partial \vartheta \partial \varphi }=[\sin\vartheta \sin\varphi ,-\sin\vartheta \cos\varphi ,0].$$

Then, the corresponding derivatives of *d_j_*(*ϑ*,*φ*) defined in Eq. (17) can be expressed as

(A6) $$\frac{\partial d_{j}}{\partial \vartheta }=\frac{\partial w}{\partial \vartheta }•p_{j}$$

(A7) $$\frac{\partial d_{j}}{\partial \varphi }=\frac{\partial w}{\partial \varphi }•p_{j}$$

(A8) $$\frac{\partial^{2}d_{j}}{\partial \vartheta^{2}}=\frac{\partial^{2}w}{\partial \vartheta^{2}}•p_{j}$$

(A9) $$\frac{\partial^{2}d_{j}}{\partial \varphi^{2}}=\frac{\partial^{2}w}{\partial \varphi^{2}}•p_{j}$$

(A10) $$\frac{\partial^{2}d_{j}}{\partial \varphi \partial \vartheta }=\frac{\partial^{2}w}{\partial \varphi \partial \vartheta }•p_{j}$$

## 8. Appendix B

Auxiliary functions defined for calculation of derivatives of *D*(*ϑ*,*φ*, ***P***_{_*_N_*_}_) defined in Eq. (28) for the directional fitting are given by:

(B1) $$a(\vartheta ,\varphi ,P_{\left\{N\right\}})=\sum_{j=1}^{N}d_{j}^{-2}$$

(B2) $$b(\vartheta ,\varphi ,P_{\left\{N\right\}})=\sum_{j=1}^{N}r_{j}d_{j}^{-1}$$

(B3) $$c_{\vartheta }(\vartheta ,\varphi ,P_{\left\{N\right\}})=\sum_{j=1}^{N}\frac{\partial d_{j}}{\partial \vartheta }d_{j}^{-3}$$

(B4) $$c_{\varphi }(\vartheta ,\varphi ,P_{\left\{N\right\}})=\sum_{j=1}^{N}\frac{\partial d_{j}}{\partial \varphi }d_{j}^{-3}$$

(B5) $$d_{\vartheta }(\vartheta ,\varphi ,P_{\left\{N\right\}})=\sum_{j=1}^{N}r_{j}\frac{\partial d_{j}}{\partial \vartheta }d_{j}^{-2}$$

(B6) $$d_{\varphi }(\vartheta ,\varphi ,P_{\left\{N\right\}})=\sum_{j=1}^{N}r_{j}\frac{\partial d_{j}}{\partial \varphi }d_{j}^{-2}.$$

Their respective derivatives are:

(B7) $$\frac{\partial a}{\partial \vartheta }=-2c_{\vartheta }$$

(B8) $$\frac{\partial a}{\partial \varphi }=-2c_{\varphi }$$

(B9) $$\frac{\partial b}{\partial \vartheta }=-d_{\vartheta }$$

(B10) $$\frac{\partial b}{\partial \varphi }=-d_{\varphi }$$

(B11) $$\frac{\partial c_{\vartheta }}{\partial \vartheta }=\sum_{j=1}^{N}\left[\frac{\partial^{2}d_{j}}{\partial \vartheta^{2}}d_{j}^{-3}-3d_{j}^{-4}{\left(\frac{\partial d_{j}}{\partial \vartheta }\right)}^{2}\right]$$

(B12) $$\frac{\partial c_{\varphi }}{\partial \varphi }=\sum_{j=1}^{N}\left[\frac{\partial^{2}d_{j}}{\partial \varphi^{2}}d_{j}^{-3}-3d_{j}^{-4}{\left(\frac{\partial d_{j}}{\partial \varphi }\right)}^{2}\right]$$

(B13) $$\frac{\partial d_{\vartheta }}{\partial \vartheta }=\sum_{j=1}^{N}\left[\frac{\partial^{2}d_{j}}{\partial \vartheta^{2}}d_{j}^{-2}-2d_{j}^{-3}{\left(\frac{\partial d_{j}}{\partial \vartheta }\right)}^{2}\right]r_{j}$$

(B14) $$\frac{\partial d_{\varphi }}{\partial \varphi }=\sum_{j=1}^{N}\left[\frac{\partial^{2}d_{j}}{\partial \varphi^{2}}d_{j}^{-2}-2d_{j}^{-3}{\left(\frac{\partial d_{j}}{\partial \varphi }\right)}^{2}\right]r_{j}$$

(B15) $$\frac{\partial c_{\vartheta }}{\partial \varphi }=\sum_{j=1}^{N}\left[\frac{\partial^{2}d_{j}}{\partial \vartheta \partial \varphi }d_{j}^{-3}-3d_{j}^{-4}\frac{\partial d_{j}}{\partial \varphi }\frac{\partial d_{j}}{\partial \vartheta }\right]$$

(B16) $$\frac{\partial d_{\vartheta }}{\partial \varphi }=\sum_{j=1}^{N}\left[\frac{\partial^{2}d_{j}}{\partial \vartheta \partial \varphi }d_{j}^{-2}-2d_{j}^{-3}\frac{\partial d_{j}}{\partial \varphi }\frac{\partial d_{j}}{\partial \vartheta }\right]r_{j}$$

where the derivatives of *d_j_* are defined in (A6-A10). Using the above functions, the derivatives of *D*(*ϑ*,*φ*, ***P***_{_*_N_*_}_) can be expressed as

(B17) $$\frac{\partial D}{\partial \vartheta }=(2bc_{\vartheta }-ad_{\vartheta })a^{-2}$$

(B18) $$\frac{\partial D}{\partial \varphi }=(2bc_{\varphi }-ad_{\varphi })a^{-2}$$

(B19) $$\begin{array}{c} \frac{\partial^{2}D}{\partial \vartheta^{2}}=a^{-2}\left(2\frac{\partial b}{\partial \vartheta }c_{\vartheta }+2b\frac{\partial c_{\vartheta }}{\partial \vartheta }-\frac{\partial a}{\partial \vartheta }d_{\vartheta }-a\frac{\partial d_{\vartheta }}{\partial \vartheta }\right)\\ -2a^{-3}\frac{\partial a}{\partial \vartheta }(2bc_{\vartheta }-ad_{\vartheta }) \end{array}$$

(B20) $$\begin{array}{c} \frac{\partial^{2}D}{\partial \varphi^{2}}=a^{-2}\left(2\frac{\partial b}{\partial \varphi }c_{\varphi }+2b\frac{\partial c_{\varphi }}{\partial \varphi }-\frac{\partial a}{\partial \varphi }d_{\varphi }-a\frac{\partial d_{\varphi }}{\partial \varphi }\right)\\ -2a^{-3}\frac{\partial a}{\partial \varphi }(2bc_{\varphi }-ad_{\varphi }) \end{array}$$

(B21) $$\begin{array}{c} \frac{\partial^{2}D}{\partial \varphi \partial \vartheta }=a^{-2}\left(2\frac{\partial b}{\partial \varphi }c_{\vartheta }+2b\frac{\partial c_{\vartheta }}{\partial \varphi }-\frac{\partial a}{\partial \varphi }d_{\vartheta }-a\frac{\partial d_{\vartheta }}{\partial \varphi }\right)\\ -2a^{-3}\frac{\partial a}{\partial \varphi }(2bc_{\vartheta }-ad_{\vartheta }) \end{array}$$

For the calculation of variances of fitted plane parameters, the following derivatives of *D*(*ϑ*,*φ*, ***P***_{_*_N_*_}_) are also needed

(B22) $$\frac{\partial D}{\partial r_{j}}=\frac{1}{d_{j}(\vartheta ,\varphi )a(\vartheta ,\varphi )}$$

(B23) $$\frac{\partial^{2}D}{\partial r_{j}\partial \vartheta }=\frac{\frac{\partial d_{j}}{\partial \vartheta }a+d_{j}\frac{\partial a}{\partial \vartheta }}{d_{j}^{2}a^{2}}$$

(B24) $$\frac{\partial^{2}D}{\partial r_{j}\partial \varphi }=-\frac{\frac{\partial d_{j}}{\partial \varphi }a+d_{j}\frac{\partial a}{\partial \varphi }}{d_{j}^{2}a^{2}}$$

## 9. Appendix C

The derivatives of the functions *A_ϑ,j_* and *A_ϕ,j_* defined in Eqs. (30a,b) and used in the gradient and Hessian calculations can be expressed as

(C1) $$\begin{array}{l} \frac{\partial }{\partial \vartheta }A_{\vartheta ,j}(\vartheta ,\varphi ,P_{\left\{N\right\}})=[d_{j}^{2}\left(d_{j}\frac{\partial^{2}D}{\partial \vartheta^{2}}-D\frac{\partial^{2}d_{j}}{\partial \vartheta^{2}}\right)\\ \;-2d_{j}^{2}\frac{\partial d_{j}}{\partial \vartheta }\frac{\partial D}{\partial \vartheta }+2d_{j}D{\left(\frac{\partial d_{j}}{\partial \vartheta }\right)}^{2}]d_{j}^{-4} \end{array}$$

(C2) $$\begin{array}{l} \frac{\partial }{\partial \varphi }A_{\varphi ,j}(\vartheta ,\varphi ,P_{\left\{N\right\}})=[d_{j}^{2}\left(d_{j}\frac{\partial^{2}D}{\partial \varphi^{2}}-D\frac{\partial^{2}d_{j}}{\partial \varphi^{2}}\right)\\ \;-2d_{j}^{2}\frac{\partial d_{j}}{\partial \varphi }\frac{\partial D}{\partial \varphi }+2d_{j}D{\left(\frac{\partial d_{j}}{\partial \varphi }\right)}^{2}]d_{j}^{-4} \end{array}$$

(C3) $$\begin{array}{l} \frac{\partial }{\partial \varphi }A_{\vartheta ,j}(\vartheta ,\varphi ,P_{\left\{N\right\}})=[\frac{\partial d_{j}}{\partial \varphi }\frac{\partial D}{\partial \vartheta }+d_{j}\frac{\partial^{2}D}{\partial \varphi \partial \vartheta }-\frac{\partial D}{\partial \varphi }\frac{\partial d_{j}}{\partial \vartheta }\\ \;-D\frac{\partial^{2}d_{j}}{\partial \varphi \partial \vartheta }-2d_{j}\frac{\partial d_{j}}{\partial \vartheta }A_{\vartheta ,j}\;]d_{j}^{-2} \end{array}$$

where the derivatives of *D*(*ϑ*, *ϕ*, ***P***_{_*_N_*_}_) and *d_j_* are calculated in Appendices A and B. For the evaluation of sensitivities of fitted plane parameters, the following derivatives are also required

(C4) $$\frac{\partial A_{\vartheta ,k}}{\partial r_{j}}=d_{k}^{-2}\left(d_{k}\frac{\partial^{2}D}{\partial r_{j}\partial \vartheta }-\frac{\partial D}{\partial r_{j}}\frac{\partial d_{k}}{\partial \vartheta }\right)$$

(C5) $$\frac{\partial A_{\varphi ,k}}{\partial r_{j}}=d_{k}^{-2}\left(d_{k}\frac{\partial^{2}D}{\partial r_{j}\partial \varphi }-\frac{\partial D}{\partial r_{j}}\frac{\partial d_{k}}{\partial \varphi }\right),$$

where the derivatives of *D* are given by Eqs. (B22–B24).

## 10. Appendix D

Using Eq. (22c), Eqs. (22a) and (22b) can be rewritten in the following form

(D1) $$\frac{\partial^{2}E_{o}}{\partial r_{j}\partial \vartheta }(\vartheta ,\varphi ,P_{\left\{N\right\}})=\frac{2}{N}[F_{\vartheta ,j}(\vartheta ,\varphi ,P_{\left\{N\right\}})-{\bar{F}}_{\vartheta }(\vartheta ,\varphi ,P_{\left\{N\right\}})]$$

(D2) $$\frac{\partial^{2}E_{o}}{\partial r_{j}\partial \varphi }(\vartheta ,\varphi ,P_{\left\{N\right\}})=\frac{2}{N}[F_{\varphi ,j}(\vartheta ,\varphi ,P_{\left\{N\right\}})-{\bar{F}}_{\varphi }(\vartheta ,\varphi ,P_{\left\{N\right\}})],$$

where

(D3) $$F_{\vartheta ,j}(\vartheta ,\varphi ,P_{\left\{N\right\}})=p_{j}•\left[\left(\frac{\partial w}{\partial \vartheta }•U_{j}\right)w+(w•U_{j})\frac{dw}{\partial \vartheta }\right]$$

(D4) $$F_{\varphi ,j}(\vartheta ,\varphi ,P_{\left\{N\right\}})=p_{j}•\left[\left(\frac{\partial w}{\partial \varphi }•U_{j}\right)w+(w•U_{j})\frac{dw}{\partial \varphi }\right],$$

and ${\bar{F}}_{\vartheta }$, ${\bar{F}}_{\varphi }$ are the averaged values

(D5) $${\bar{F}}_{\vartheta }(\vartheta ,\varphi ,P_{\left\{N\right\}})=\frac{1}{N}\sum_{k=1}^{N}F_{\vartheta ,k}(\vartheta ,\varphi ,P_{\left\{N\right\}})$$

(D6) $${\bar{F}}_{\varphi }(\vartheta ,\varphi ,P_{\left\{N\right\}})=\frac{1}{N}\sum_{k=1}^{N}F_{\varphi ,k}(\vartheta ,\varphi ,P_{\left\{N\right\}}).$$

Equations (D1) and (D2) show that the components of the vector ***V****_j_* in Eq. (13b) applied to *E_O_* depend explicitly on the measured range *r_j_*.

Equations (33a) and (33b) can be rewritten in the following form

(D7) $$\begin{array}{l} \frac{\partial^{2}E_{D}}{\partial r_{j}\partial \vartheta }(\vartheta ,\varphi ,P_{\left\{N\right\}})=\\ \frac{2}{N}\left\{\sum_{k=1}^{N}\left[A_{\vartheta ,k}\frac{\partial D}{\partial r_{j}}d_{k}^{-1}+\left(\frac{D}{d_{k}}-1\right)\frac{\partial A_{\vartheta ,k}}{\partial r_{j}}\right]-A_{\vartheta ,j}\right\} \end{array}$$

(D8) $$\begin{array}{l} \frac{\partial^{2}E_{D}}{\partial r_{j}\partial \varphi }(\vartheta ,\varphi ,P_{\left\{N\right\}})=\\ \frac{2}{N}\left\{\sum_{k=1}^{N}\left[A_{\varphi ,k}\frac{\partial D}{\partial r_{j}}d_{k}^{-1}+\left(\frac{D}{d_{k}}-1\right)\frac{\partial A_{\varphi ,k}}{\partial r_{j}}\right]-A_{\varphi ,j}\right\}. \end{array}$$

From the Eqs. (C4), (C5), and (B22) it follows that none of the derivatives $\frac{\partial A_{\vartheta ,k}}{\partial r_{j}}$, $\frac{\partial A_{\varphi ,k}}{\partial r_{j}}$, or $\frac{\partial D}{\partial r_{j}}$ depends on *r_j_*. In fact, a measured range *r_j_* enters the right hand side of Eqs. (D7) and (D8) only indirectly in the formula for *D* and its angle derivatives; see Eqs. (28), (30a,b), (B17) and (B18). Thus, the influence of a particular *j*-th range measurement on the components of the vector ***V****_j_* in Eq. (13b) applied to *E_D_* is negligible for typical datasets with large *N*.
